# The DSF Family of Cell–Cell Signals: An Expanding Class of Bacterial Virulence Regulators

**DOI:** 10.1371/journal.ppat.1004986

**Published:** 2015-07-16

**Authors:** Robert P. Ryan, Shi-qi An, John H. Allan, Yvonne McCarthy, J. Maxwell Dow

**Affiliations:** 1 Division of Molecular Microbiology, College of Life Sciences, University of Dundee, Dundee, United Kingdom; 2 School of Microbiology, Biosciences Institute, University College Cork, Cork, Ireland; University of Basel, SWITZERLAND

## Abstract

Many pathogenic bacteria use cell–cell signaling systems involving the synthesis and perception of diffusible signal molecules to control virulence as a response to cell density or confinement to niches. Bacteria produce signals of diverse structural classes. Signal molecules of the diffusible signal factor (DSF) family are *cis*-2-unsaturated fatty acids. The paradigm is *cis*-11-methyl-2-dodecenoic acid from *Xanthomonas campestris* pv. *campestris (Xcc)*, which controls virulence in this plant pathogen. Although DSF synthesis was thought to be restricted to the xanthomonads, it is now known that structurally related molecules are produced by the unrelated bacteria *Burkholderia cenocepacia* and *Pseudomonas aeruginosa*. Furthermore, signaling involving these DSF family members contributes to bacterial virulence, formation of biofilms and antibiotic tolerance in these important human pathogens. Here we review the recent advances in understanding DSF signaling and its regulatory role in different bacteria. These advances include the description of the pathway/mechanism of DSF biosynthesis, identification of novel DSF synthases and new members of the DSF family, the demonstration of a diversity of DSF sensors to include proteins with a Per-Arnt-Sim (PAS) domain and the description of some of the signal transduction mechanisms that impinge on virulence factor expression. In addition, we address the role of DSF family signals in interspecies signaling that modulates the behavior of other microorganisms. Finally, we consider a number of recently reported approaches for the control of bacterial virulence through the modulation of DSF signaling.

## Introduction

**Fig 1:**
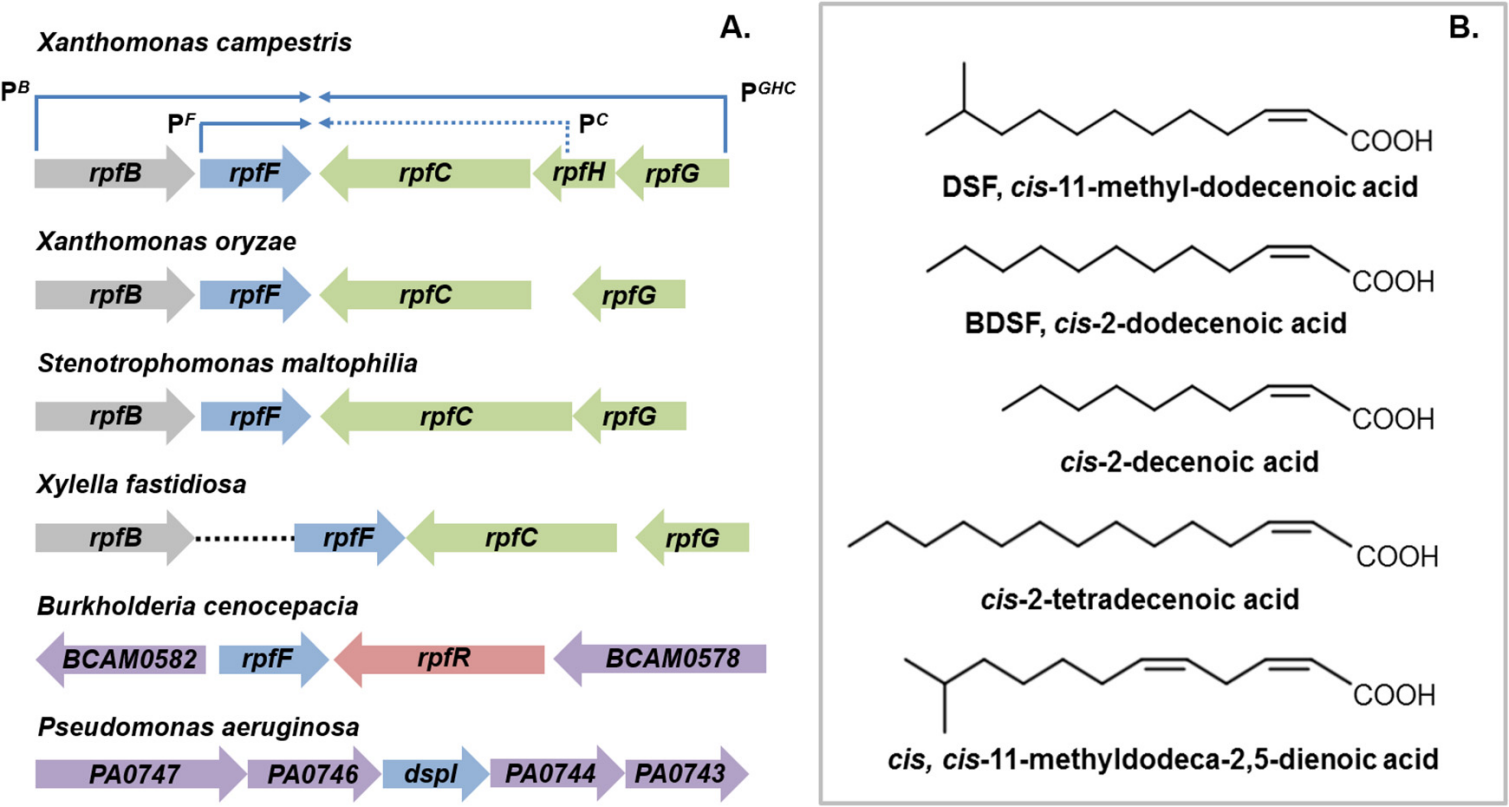
DSF family signals and the organization of *rpf* gene clusters that direct signal synthesis and perception. **(A)** RpfF, an enzyme of the crotonase superfamily, is involved in synthesis of DSF in all bacteria. Two core pathways of DSF perception have been described: (1) the RpfCG two-component system first identified in xanthomonads and (2) RpfR, a protein with PAS, GGDEF, and EAL domains, first identified in *Burkholderia* species. In *Xanthomonas campestris*, the genes encoding RpfC and RpfG are organized in an operon that is convergently transcribed to *rpfF* (top). This operon also contains *rpfH*, a gene encoding a protein similar to the input domain of RpfC but of no known function. The *rpfF* gene is found in an operon with *rpfB*, which encodes a fatty acyl CoA ligase but also has its own promoter. This organization of *rpf* genes occurs in all xanthomonads with the variations that *rpfH* is not widely conserved and *rpfB* can be located in a different genomic location, as is seen in *Xylella fastidiosa*. In *Burkholderia* species, *rpfF* is convergently transcribed with *rpfR*, which encodes the sensor protein. In *Pseudomonas aeruginosa*, the *dspI* gene that encodes an RpfF homolog is located in a cluster of genes encoding enzymes implicated in fatty acid metabolism. The identity of the sensor for the signal is not known. **(B)** The DSF family of signals comprises *cis*-2-unsaturated fatty acids of different chain lengths and branching. The paradigm *cis*-11-methyl–dodecenoic acid designated DSF, was first described in *Xanthomonas campestris*. Different signals were then described in *Burkholderia cenocepacia* (BDSF), *Pseudomonas aeruginosa* (*cis*-2-decenoic acid), *Xylella fastidiosa* (*cis*-2-tetradecenoic acid) and *Xanthomonas oryzae* (*cis*,*cis*-11 methyldodeca-2,5-dienoic acid). It is now established that each of these bacteria produces multiple DSF family signals, although each genus seems to be most responsive to the major signal that it produces. DSF family signals can be involved in bi-directional interspecies signaling. However unidirectional signaling is evident; *Pseudomonas aeruginosa* responds to DSF and BDSF produced by xanthomonads, but these latter organisms do not respond to *cis*-2-decenoic acid, the *P*. *aeruginosa* signal.

The discovery of diffusible signal factor (DSF) signaling arose from a molecular genetic analysis of the regulation of the synthesis of virulence factors in the plant pathogen *Xanthomonas campestris* pv. *campestris* (*Xcc*) [1,2]. These studies identified a cluster of genes called *rpf* (standing for “regulation of pathogenicity factors”), mutation of which leads to coordinate down-regulation of the synthesis of a number of extracellular enzymes (including endoglucanase and protease) and the extracellular polysaccharide xanthan as well as reduced virulence to plants [1,2]. Subsequent work revealed that the products of several of these *rpf* genes were involved in a signaling system involving synthesis and perception of a diffusible molecule that was called DSF, for diffusible signal factor [2,3]. The synthesis of DSF in *Xcc* is totally dependent on RpfF, which has amino acid sequence relatedness to enoyl CoA hydratase and is partially dependent on RpfB, which is a long-chain fatty acyl CoA ligase. DSF sensing and signal transduction involves a two-component system comprising the sensor RpfC and regulator RpfG [3]. These proteins are encoded by the *rpfGHC* operon, which is adjacent to *rpfF* and convergently transcribed (Fig 1A). Perception of DSF by RpfC is linked to phosphorylation of the HD-GYP domain regulator RpfG and alteration in the cellular level of the second messenger cyclic di-GMP [3,4]. Different pathways then act to control different sub-sets of Rpf-regulated virulence functions. RpfC acts to positively regulate synthesis of virulence factors, but to negatively regulate DSF synthesis. The elevated level of DSF in *rpfC* mutants allowed characterization of the signal as the unsaturated fatty acid *cis*-11-methyl-dodecenoic acid (Fig 1B), representing the paradigm for a novel structural class of signal [3,5]. The *cis* unsaturated double bond at the 2-position is a key structural feature for activity; this structural motif is regarded as the signature for DSF family signals [5].

Comparative genomic studies have indicated conservation of clustered *rpfF*, *rpfG*, and *rpfC* genes, and by extension DSF-mediated signaling, throughout the xanthomonads [6]. Furthermore, DSF synthesis and signaling is known to influence the virulence of several *Xanthomonas* spp. and *Xylella fastidiosa*, which are plant pathogens, and *Stenotrophomonas maltophilia*, some strains of which are opportunistic human pathogens [7,8]. In most of these bacteria, DSF acts to positively influence virulence, whereas in *Xylella fastidiosa*, DSF-deficient mutants show enhanced virulence [7,8]. *Xylella fastidiosa* is transmitted between plants exclusively by xylem sap-feeding insects. Intriguingly, DSF also influences bacterial interactions with these vectors. DSF-deficient mutants have a reduced capacity to colonize their insect vector and to form biofilms in the insect foregut [8]. This reduced retention leads to poor transmission to uninfected plants [8].

DSF synthesis was originally thought to be restricted to the xanthomonads and to unrelated bacteria with an *rpfF*-*rpfG*-*rpfC* gene cluster, such as *Methylophaga*, *Leptospirillum*, *Thiobacillus*, and *Methylobacillus* species [9]. Later findings, however, suggested a much broader significance for DSF signaling in the bacterial world. Structurally related molecules are produced by the unrelated bacteria *Burkholderia cenocepacia* and *Pseudomonas aeruginosa*: these molecules are *cis-2-*dodecenoic acid (BDSF) and *cis-*2-decenoic acid, respectively (Fig 1B) [10,11]. Signaling involving these DSF family members contributes to bacterial virulence, formation of biofilms, and antibiotic tolerance in these important human pathogens [11,12].

In addition to intraspecies signaling, DSF family signals have been implicated in interspecies and interkingdom signaling, in which they modulate the behavior of other microorganisms that do not produce the signal. For example, although *Pseudomonas aeruginosa* does not synthesize DSF or BDSF, it is capable of sensing these molecules, with consequences for bacterial behavior [9]. The dimorphic fungus *Candida albicans* can also respond to BDSF to modulate the yeast-hyphal transition [10].

Over the last five years, significant progress has been made in our understanding of DSF-family signaling. These advances have included (i) the discovery of the biosynthetic pathway/mechanism for DSF; (ii) the identification of new DSF family signals in a variety of additional bacterial pathogens; (iii) the discovery of alternate mechanisms for the sensing of DSF family signals that involved diverse proteins with PAS (Per-Arnt-Sim) domains; (iv) advances in the mechanistic understanding of the signal transduction pathways that follow DSF perception and their role in regulation of bacterial virulence; and (v) several seminal studies that demonstrate that modulation of DSF signaling may have potential in controlling bacterial disease. Here, we review these recent insights into the broad significance of DSF-family signaling systems in bacteria. We conclude by identifying some outstanding research questions concerning this fascinating family of signal molecules and their role in plant and animal pathogenic bacteria.

## The Role of RpfF and RpfB in DSF Biosynthesis and Signaling

**Fig 2:**
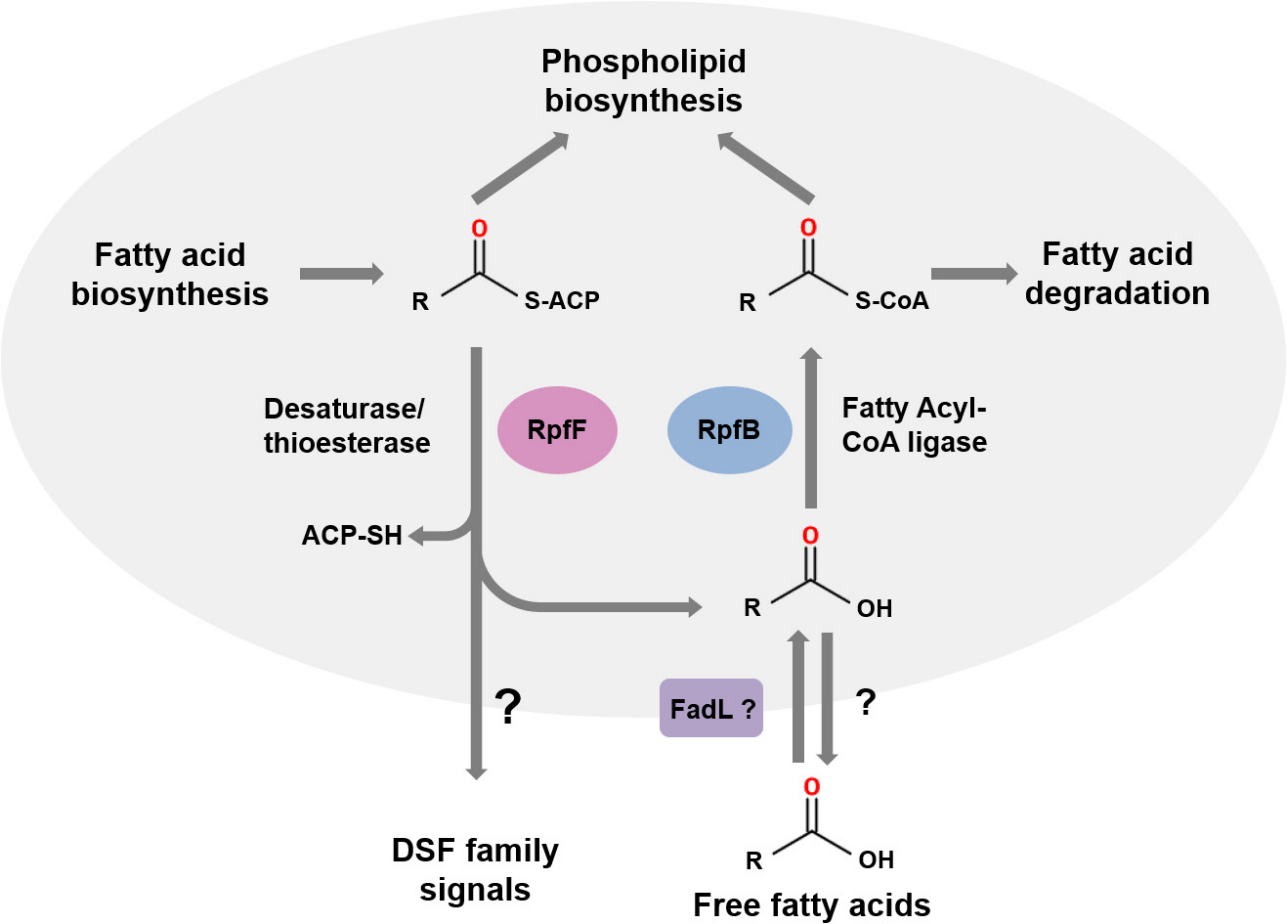
The roles of RpfF and RpfB in DSF signaling. RpfF encodes an enzyme from the crotonase family that possesses both desaturase and thioesterase activities and uses fatty acyl ACP substrates derived from fatty acid biosynthesis as substrates. DSF family signals are generated from 3-hydroxy-fatty acyl ACPs by consecutive or coordinated desaturase and thioesterase action. In addition, the thioesterase activity of RpfF generates a range of free hydroxylated and non-hydroxylated fatty acids. DSF family signals leave the cell by as-yet-unknown efflux mechanisms (indicated by the question mark). Free fatty acids whose synthesis depends upon RpfF can also be found in the culture supernatant. The role of RpfB is in mobilization of these free fatty acids. Uptake, which may involve FadL, is followed by conversion to fatty acyl CoA derivatives that can be used in phospholipid biosynthesis or for degradation. This figure is a modified version of that published by Bi and colleagues [15].

RpfF proteins have amino acid sequence similarity to enoyl CoA hydratases, which are members of the crotonase superfamily of enzymes [6,10]. Recent studies of BCAM0581, a homolog of RpfF, from *B*. *cenocepacia* have identified the immediate substrate(s) and some aspects of the enzymatic mechanism of these DSF synthases [13]. In vitro, BCAM0581 utilizes a 3-hydroxylated fatty acyl-ACP (acyl carrier protein) as substrate rather than a CoA derivative and through its desaturase and thioesterase activity generates the *cis*-2-unsaturated fatty acid BDSF (Fig 2). The current model for the action of BCAM0581 is that the enzyme first works as a dehydratase to convert 3-hydroxydodecanoyl-ACP to *cis*-2-dodecenoyl-ACP and then as a thioesterase to release free BDSF (*cis*-2-dodecenoic acid) [13]. The mechanistic details underpinning these two actions, which may be coupled in vivo, are unclear. RpfF is the only member of the crotonase superfamily with both desaturase and thioesterase activity. In vivo, the substrates for RpfF are presumably intermediates in fatty acid biosynthesis. Because RpfF has thioesterase activity, it can generate free saturated fatty acids from any fatty acyl ACP substrate. In the in vitro assay, an exogenous acyl-ACP synthetase is added to reverse this thioesterase reaction, promoting BDSF synthesis. Although RpfB was originally thought to be involved in DSF synthesis or processing [2,14], new work has established that it has a different role, acting in the mobilization of (saturated) free fatty acids generated by the thioesterase action of RpfF [15]. In *Xcc*, RpfB, which is a predicted fatty acid CoA ligase, activates these free fatty acid derivatives so that they can be used in phospholipid biosynthesis (Fig 2). In this way, RpfB counteracts the thioesterase activity of RpfF. RpfB has little activity against BDSF, however. Orthologs of RpfB occur widely in *Burkholderia* spp., although the encoding genes are not linked to *rpfF*, in contrast to what is seen in most xanthomonads (Fig 1A).

The dual nature of BCAM0581 as a broad specificity thioesterase as well as a desaturase is also seen in vitro with the *Xcc* RpfF enzyme and is consistent with a number of previous observations on effects of *rpfF* mutation on fatty acid profiles in culture supernatants [16,17]. Loss by mutation of RpfF in *S*. *maltophilia* affects synthesis of not only DSF but also seven structurally related saturated and unsaturated fatty acids [17]. Culture supernatants of *X*. *fastidiosa* contain 12-methyl-tetradecanoic acid [18], as well as the authentic DSF-family signal *cis*-2-tetradecenoic acid, a recently described member of the DSF family (see Fig 1B) [19,20].

Individual bacteria can produce multiple DSF family signals. For example, bacteria within the *Burkholderia cepacia* complex (Bcc) as well as *Xanthomonas* spp. synthesize DSF, BDSF, and *cis*,*cis*-11-methyldodeca-2,5-dienoic acid (Fig 1B) [21–23]. These multiple DSF family signals are all dependent on RpfF for their synthesis. Swapping RpfF homologs between bacteria in the Bcc complex indicates that the pattern of DSF, BDSF, and *cis*,*cis*-11-methyldodeca-2,5-dienoic acid signals produced is regulated by the supply of different substrates, rather than differences in specificity of different RpfF synthases [22]. Consistent with this contention, in *X*. *oryzae* pv. *oryzae*, the rice pathogen, the three signals are present in different ratios depending on the culture medium and are produced with different time courses during growth [23]. The substrate for DSF synthesis in vivo must be 11-methyl-3-hydroxydodecanoyl-ACP, with the 11-methyl substitution presumably derived from leucine via the branched chain fatty acid synthetic pathway. Whether production of multiple DSF-family signals by one organism has any biological relevance is unclear. However the systems for sensing DSF family signals within a particular organism appear to be attuned to the major signal produced by that organism. For example, *Xcc* and *X*. *fastidiosa* generate *cis*-11-methyl-dodecenoic acid and *cis*-2-tetradecenoic acid respectively via highly related RpfF proteins, but each is more responsive to its own signal than the heterologous one [23].

## The Interaction of RpfF and RpfC and Signal Transduction

As outlined above, RpfC in *Xcc* acts as a sensor for DSF but also in repression of DSF biosynthesis. DSF sensing and signal transduction leading to activation of the production of extracellular enzymes and EPS requires conserved amino acid residues of RpfC implicated in phosphorelay. In contrast, RpfC repression of DSF synthesis does not require phosphorelay but is mediated instead by protein–protein interactions between the REC domain of RpfC and RpfF, the DSF synthase [24]. Sequestration of RpfF in this manner may restrict synthesis of DSF; loss of RpfC by mutation results in a highly elevated level of DSF [24]. Release of RpfF as a result of conformational changes in RpfC that occur upon DSF binding has been invoked as a mechanism to allow rapid auto-induction of DSF synthesis [24,25]. However, subsequent experimental evidence indicates that DSF synthesis in *Xcc* is not auto-inductive, suggesting that this may not be the true role for this interaction [26]. Recent work in *X*. *fastidiosa* has provided potential further insight into the role for RpfF–RpfC interactions [26]. In *Xcc*, the synthesis of extracellular enzymes and EPS in an *rpfF* mutant can be restored to wild type by exogenous DSF. By contrast, addition of the *Xylella* DSF family signal does not restore the phenotype of the *rpfF* mutant to wild type. Instead DSF signal transduction in *Xylella* requires both RpfC and RpfF [26]. Enzymatically inactive variants of RpfF in which two conserved catalytic glutamate residues are replaced by alanine residues can also support DSF signal transduction. These findings reveal RpfF in *Xylella* to be a multifunctional protein. A plausible model for RpfF action is that it can interact with multiple REC domain proteins, and release from RpfC upon binding of DSF (or other signals) allows its interaction with these other regulators, thereby effecting signal transduction through “partner swapping.

Intriguingly, analysis of a panel of clinical isolates of *S*. *maltophilia* revealed distribution of two sub-groups of *rpf* gene cluster that were associated with different levels of DSF synthesis and virulence regulation [27]. Two populations of RpfF called RpfF-1 and RpfF-2 were distinguished by differences in the N-terminal region of the protein. Each RpfF variant is associated with a specific RpfC variant (RpfF1 with RpfC1 and RpfF2 with RpfC2). RpfC1 and RpfC2 differ in the number of transmembrane helices in the sensory input domain [27]. Only RpfC-1-RpfF-1 variant strains display detectable DSF production; the observed DSF-deficient phenotype of RpfC-2-RpfF-2 variant strains is due to permanent repression of RpfF-2 by RpfC-2.

## Expansion of Classes of Sensor Involved in DSF Signal Perception

**Fig 3:**
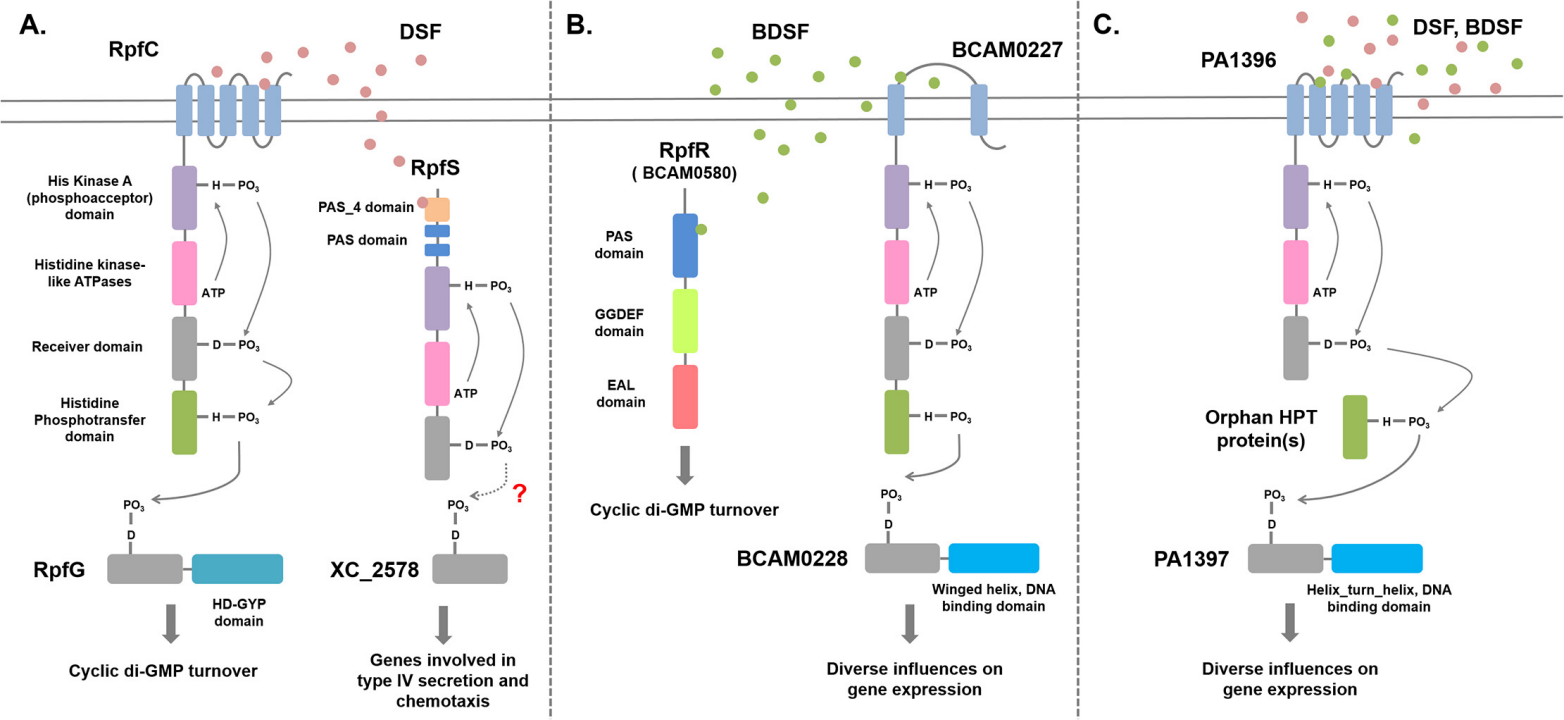
Signal transduction mechanisms for DSF family signals in different bacteria. **(A)** *Xanthomonas campestris*. DSF perception involves the complex histidine kinase RpfC. Binding of DSF to the sensory input domain, which comprises five transmembrane helices, leads to autophosphorylation, phosphorelay via the receiver and histidine phosphotranfer domains, and phosphotransfer to the receiver domain of the response regulator RpfG. RpfG has an HD-GYP domain, which is a cyclic di-GMP phosphodiesterase; phosphorylation activates RpfG for cyclic di-GMP degradation. A second sensing system for DSF involves the soluble histidine kinase RpfS, which binds DSF through the N-terminal PAS_4 domain. RpfS influences the expression of a sub-set of DSF-regulated genes, in particular those involved in the epiphytic growth phase. This action is likely exerted through the response regulator XC_2578. The RpfGC system is “core,” being found in all xanthomonads, including *Xylella fastidiosa*, whereas RpfS is “accessory” and is not fully conserved. In *Xylella*, DSF signal transduction also involves RpfF (see text for details). **(B)** *Burkholderia cenocepacia*. BDSF perception involves the soluble PAS-GGDEF-EAL domain protein RpfR. Binding of BDSF to the N-terminal PAS domain activates RpfR for cyclic di-GMP degradation by the cyclic di-GMP phosphodiesterase EAL domain. A second sensing system for BDSF involves the histidine kinase BCAM0227, which differs from RpfC in having two transmembrane helices and a large periplasmic domain. Signal transduction from BCAM0227 involves autophosphorylation upon BDSF binding, phosphorelay, and phosphotransfer to the DNA-binding regulator BCAM0228. The RpfFR system is “core,” being found in widely in *Burkholderia* and other genera, whereas BCAM0227 is “accessory” and is only found in strains of *B*. *cenocepacia*. **(C)** Interspecies signaling in *Pseudomonas aeruginosa*. BDSF or DSF perception involves the membrane-associated histidine kinase PA1396, which resembles RpfC but does not have an HPt domain. Signal transduction from PA1396 involves autophosphorylation upon BDSF binding, phosphorelay, and phosphotransfer via an orphan HPt domain protein to the DNA-binding regulator PA1397. This system influences the expression of genes involved in polymyxin resistance and stress tolerance.

The discovery of BDSF synthesis in *B*. *cenocepacia* through the action of the RpfF ortholog BCAM0581 raised the issue of the mechanisms by which the signal is perceived [10]. Two sensors for BDSF have now been described; BCAM0580, which is designated RpfR, and the sensor kinase BCAM0227 [28,29]. RpfR (see Fig 3) comprises an N-terminal PAS domain and GGDEF and EAL domains. GGDEF and EAL domains are implicated in the synthesis and degradation, respectively, of the second messenger cyclic di-GMP [29,30]. In vitro, RpfR exhibits cyclic di-GMP phosphodiesterase activity that is modulated by binding of BDSF to the N-terminal PAS domain [29]. Thus, in both *B*. *cenocepacia* and *Xcc*, sensing of a DSF family signal is linked to cyclic di-GMP turnover, but the mechanisms are completely different (Fig 3). Notably, this was the first description of the role of a PAS domain in sensing DSF-family signals. Bioinformatic analysis reveals that the RpfR-RpfF system is widely conserved not only in *Burkholderia* species but also in bacteria from related genera, such as *Achromobacte*r, and unrelated Enterobacteriacaeae, including *Yersinia*, *Serratia*, *Cronobacter*, and *Enterobacter*.

BCAM0227 is a complex sensor kinase but is not a homolog of RpfC, the DSF sensor in *Xcc* [28]. BCAM0227 is predicted to have two transmembrane helices and a large periplasmic loop of 300 amino acids, whereas RpfC is predicted to have five transmembrane helices (Fig 3). Comparative transcriptome analysis showed that BCAM0227 is involved in regulation of a subset of functions that are controlled by BDSF in *B*. *cenocapacia* [28]. Unlike RpfR, BCAM0227 is restricted to *B*. *cenocepacia* [29]. This may suggest that RpfR-RpfF is a second “core” system for DSF signaling in addition to RpfF-RpfC-RpfG of the xanthomonads, whereas BCAM0227 is an accessory sensor.

Alternative sensors for DSF family signals have also been proposed for *Xanthomonas* spp and *Xylella* [31–33]. These suggestions were based on comparative transcriptome analysis of wild type and *rpfF*, *rpfC*, and *rpfG* mutants [31–33]. The effects of these mutations were not consistent with the existence of a single linear pathway, and pointed to the occurrence of alternative sensors for DSF and alternative regulators that interact with RpfC [31–34]. A genetic screen identified XC_2579 (RpfS) as a second sensor for DSF in *Xcc* [35]. RpfS is a histidine sensor kinase with an N-terminal PAS domain (PAS_4 domain) that binds DSF and is required for regulation (Fig 3). RpfS controls expression of a specific subset of genes controlled by DSF and has a role in the epiphytic phase of the disease cycle [35]. Homologs of RpfS occur in many, but not all, *Xanthomonas* and *Stenotrophomonas* species, suggesting that it is an accessory element in DSF signaling.

PAS domains are known to be highly divergent at the primary sequence level, but nevertheless have a conserved three-dimensional architecture within which structural clades can be discerned [36]. This structural division of PAS domains may reflect, in part, the class of small molecule that they bind. The PAS domains of XC_2579 and RpfR lack significant sequence similarity, and further work will be needed to show if they are nonetheless highly related, structurally.

## DSF Signaling and the Regulation of Bacterial Virulence, Biofilm Formation, and Antibiotic Resistance

In many bacteria, DSF family-mediated signaling contributes to bacterial virulence, formation of biofilms and antibiotic tolerance; examples are given in Table 1. Perception of DSF-family signals by the two “core” pathways is linked to cyclic di-GMP turnover. This second messenger interacts with a range of effectors to exert a regulatory action at transcriptional, post-transcriptional, and post-translational levels [30]. Hence, identifying factors under DSF control has required transcriptomic and proteomic approaches as well as assessment of phenotypes [31–34,37–39].

**Table 1:** Virulence-related and other factors that are regulated by DSF family signals in diverse pathogens.

Organism	Biological response	Reference
**DSF family signals in intraspecies signaling**		
*Xanthomonas campestris*	Extracellular enzyme production; extracellular polysaccharide production; PilA-dependent motility; biofilm dispersal; small RNA synthesis; elongation factor P turnover; cyclic glucan synthesis; *Arabidopsis* stomatal opening factor(s)	[2,33,38,55–58]
*Xanthomonas oryzae* pvs. *oryzae* and *oryzicola*	Iron uptake; iron-dependent virulence to rice; extracellular proteases; extracellular polygalacturonase, asparagine synthase	[39,56,59,60]
*Xanthomonas axonopodis*	Extracellular enzyme production; extracellular polysaccharide production; flagellar-dependent biofilm formation	[61,62]
*Xanthomonas citri* subsp. *citri*	Host leaf surface adherence and penetration in lemon and grapefruit leaves	[37]
*Xanthomonas hortorum* pv. *pelargonii*	Virulence; in planta movement	[63]
*Xylella fastidiosa*	Insect vector colonization and attachment; transmission to plants; outer membrane vesicle release; extracellular enzyme production	[8,64,65]
*Pseudomonas aeruginosa*	Biofilm dispersal; reversal of dormant persister cell state	[11,40,66]
*Burkholderia cenocepacia*	Adherence, biofilm formation and swarming motility; virulence in mouse, zebrafish and insect models	[28,29,67]
*Stenotrophomonas maltophilia*	Heavy metal tolerance and antibiotic resistance; extracellular protease production; virulence to *Galleria mellonella* larvae	[16]
**DSF family signals in interspecies and interkingdom signaling**		
*Pseudomonas aeruginosa*	Polymyxin resistance; biofilm formation; persistence in mouse models; type III secretion	[9,42]
*Candida albicans*	Inhibition of yeast-hyphal dimorphic transition; adherence to catheters	[10,68–70]
*Staphylococcus aureus*	Inhibition of aminoglycoside resistance; inhibition of biofilm formation	[41,50]
*Bacillus cereus*	Inhibition of biofilm formation; susceptibility to aminoglycosides	[41,50]
*Escherichia coli*	Inhibition of biofilm formation; revert persister cells to susceptible state	[11,41]

## DSF Family Signals and Intra- and Inter-species Signaling in *Pseudomonas aeruginosa*

**Fig 4:**
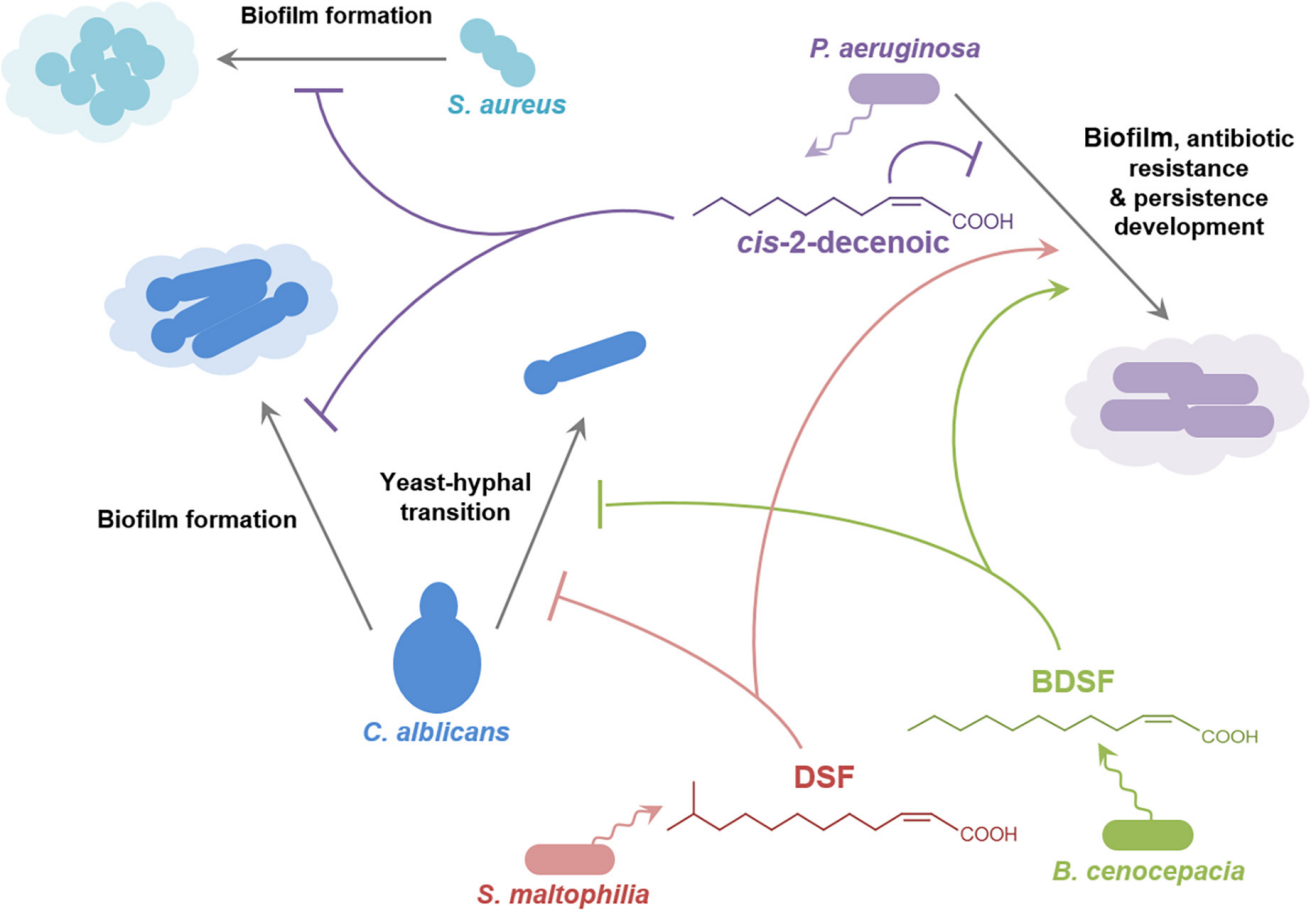
Signals of the DSF family in interspecies and inter-kingdom signaling. DSF family signals produced by *Stenotrophomonas maltophilia* (principally DSF) and *Burkholderia cenocepacia* (principally BDSF) influence the behavior of *Pseudomonas aeruginosa* and the yeast–hyphal transition in *Candida albicans*. The *P*. *aeruginosa* signal *cis*-2-decenoic acid prevents biofilm by *Candida albicans* and growth and biofilm formation by *Staphylococcus aureus*. The effects of DSF and BDSF on *Pseudomonas aeruginosa* are exerted via the sensor kinase PA1396, which does not respond to *cis*-2-decenoic. The production of DSF and BDSF may allow interspecies communication between *Stenotrophomonas maltophilia* and *Burkholderia cenocepacia*. Other signal molecules are also involved in interaction between some of these organisms, but are not indicated here for clarity. For example, 3-oxo-dodecanoyl-homoserine lactone produced by *P*. *aeruginosa* can influence *C*. *albicans* and *B*. *cenocepacia*.

Studies in *P*. *aeruginosa* have provided an insight into signaling involving the DSF family through identification of new members of the family (*cis*-2-decenoic acid; Fig 1) [11], and the role that DSF has in interspecies communication (Fig 4) [9,40]. *Cis*-2-decenoic acid was identified as a factor produced by *P*. *aeruginosa* that can induce dispersion of biofilms produced by *P*. *aeruginosa* as well as other bacteria, and inhibit the growth of *Staphylococcus aureus* (Fig 4) [11,41]. An RpfF homolog called DspI (PA0745) is required for production of *cis*-2-decenoic acid [40]. The action of the purified DspI protein has not been tested, but is inferred from the effects of mutation of the *dspI* gene on the production of *cis*-2-decenoic acid in the culture medium [40]. DspI contains 15 out of the 29 conserved amino acids of the predicted ligand-binding site of RpfF from *Xanthomonas oryzae* [40] and has the two conserved glutamates known to be essential for DSF production by RpfF from *Xanthomonas campestris* and *Xylella fastidiosa* [25,26], suggesting that it acts by the same mechanism. Intriguingly, *PA0745* is located in a putative operon with genes encoding other fatty acid–handling enzymes including an acyl-CoA dehydrogenase, a 3-hydroxyisobutyrate dehydrogenase and a putative enoyl-CoA hydratase/isomerase. Homologs of DspI occur in over ten *Pseudomonas* species, suggesting that production of DSF family signal molecules may be widespread among members of the genus [40]. *Cis*-2-decenoic acid does not effectively activate the DSF signaling pathway in *Xcc*, but conversely, DSF and BDSF can activate changes in gene expression, alter biofilm formation, and increase antibiotic tolerance in *P*. *aeruginosa* [9,30]. This action is exerted via PA1396, a sensor kinase with an input domain very similar to that of RpfC of *Xcc* [9]. Such interspecies signaling may occur in multispecies infections, such as those associated with cystic fibrosis (CF) in which *P*. *aeruginosa* is present together with *S*. *maltophilia* and *Burkholderia* species (Fig 4) [42]. The detection of DSF and BDSF signals at physiologically relevant levels in the sputum of CF patients supports this contention [42]. PA1396 does not respond to *cis*-2-decenoic acid, however, so the identity of the sensor for this intra-species signal remains obscure.

## The Role of DSF Family Signals in Interkingdom Signaling

The DSF family of signal molecules has been implicated in inter-kingdom signaling between bacteria and the dimorphic fungal pathogen *C*. *albicans*. DSF, BDSF, and CDSF can all act to modulate the morphological yeast-hyphal transition of *C*. *albicans* (Fig 4) [10,43]. Such interactions may be important in the CF airway, in which *C*. *albicans* can be part of the polymicrobial community. A range of other compounds able to inhibit the *Candida* yeast–hyphal transition include the fungal signals farnesol and farnesoic acid, dodecanol, the bacterial quorum sensing signal 3-oxo-C12-homoserine lactone and *trans*-2-decenoic acid produced by *Streptococcus mutans* [44]. It is not known if all these molecules exert their action through the same mechanism.

There is an expanding literature on the influence that bacterial signal molecules such as *N*-acyl homoserine lactones and the *Pseudomonas* quinolone signal (PQS) can have on mammalian host cells [44,45]. In contrast, there is, as yet, almost no information on the action of DSF family molecules on plant or mammalian hosts. Examination of the effects of DSF on the salicylic acid and abscisic acid signaling pathways in rice revealed little impact on marker genes for the former and only a minor suppressive effect on one marker gene for the latter [46].

## Establishment of Methods for Interference with DSF Family Signaling As a Route to Control Bacterial Disease

The role of DSF signaling in regulation of virulence factors has indicated that it may be a potential target for interference to allow control of disease. Work on the behavior of *X*. *fastidiosa* and *Xcc* has indicated that DSF signaling is normally finely balanced during the disease process and that such a fine balance might therefore be readily disrupted by either degradation or over-production of the signal, with consequences for disease control [31]. One approach has been to engineer plants to express RpfF and hence elevate DSF levels [47,48]. Plant expression of RpfF from *X*. *fastidiosa* in citrus and grape reduces the virulence of the important plant pathogens *X*. *citri* and *X*. *fastidiosa* respectively [47,48]. Although the synthesis of virulence factors in *X*. *citri* within transgenic citrus is reduced, the underlying mechanisms are not clear. It is plausible that synthesis of DSF activates plant defense responses that impair bacterial growth and gene expression. Alternatively, expression of RpfF may generate DSF and/or structural analogs that directly influence cell-cell signaling. Expression of the *Xylella* RpfF in grape conferred the production of not only *cis*-2-tetradecenoic acid (the *Xylella* signal) but also DSF and *cis*-2-hexadecenoic acid, two compounds that have not been found in cultures of *X*. *fastidiosa* [19].

Inoculation of bacteria able to degrade DSF can reduce virulence and symptom production by *X*. *fastidiosa* in grape and *Xcc* in brassica [49]. This suggests that it should be possible to select or engineer strains for use as biocontrol agents for plant diseases. The mechanisms by which bacteria degrade or inactivate DSF are unknown, although rapid degradation or inactivation of DSF by a *Pseudomonas* spp strain G requires *carAB*, which encode enzymes responsible for the synthesis of carbamoylphosphate, a precursor in biosynthesis of pyrimidines and arginine [49].

To influence disease in animals or humans, a rational approach would be the identification of molecules that block key signal sensing or transduction steps. Structural analogues of DSF may have a role in control of virulence factor synthesis in different pathogens. Such molecules may represent lead compounds for new drugs. The signal molecules themselves may also be of use. BDSF can act to enhance the antimicrobial efficacy of antibiotics against some bacterial pathogens and inhibit *C*. *albicans* adherence to catheters [50,51]. *Cis*-2-decenoic acid can inhibit growth and biofilm formation by *S*. *aureus* [41], in combination with antibiotics can eradicate pre-established biofilms of a number of bacteria [52,53], and can revert antimicrobial-insensitive persister cells of *P*. *aeruginosa* and *Escherichia coli* to a susceptible state [54]. Although such approaches discussed above are still in their infancy, the examples outlined in combination with other control measures could facilitate improved approaches to prevent and treat bacterial infections associated with a range of plant, animal, and human diseases.

## Concluding Remarks

It is now evident that signal molecules of the DSF family play a significant role in regulation of virulence and disease progression in a wide range of plant, animal, and human bacterial pathogens. However, the recent advances outlined above pose a new set of outstanding research questions. Will the determination of the structure of RpfF from both *Xanthomonas* and *Stenotrophomonas* species aid in defining the mechanistic details of DSF synthesis and allow the rational development of inhibitors? Will analysis of PAS domains identify structural features specifying DSF binding? The expansion of the classes of DSF receptor suggests that further DSF-family–mediated intra-species or inter-species signaling remains to be predicted and identified. How does the *P*. *aeruginosa* signal-response network involve *cis*-2-decenoic function? Will this represent a third class of widely conserved DSF-family signaling system? What are the actions of DSF family signals on fungi other than *C*. *albicans* and on eukaryotic hosts of plant and animal pathogens? Unquestionably, future research to address these issues will most likely uncover further fascinating aspects of this family of signal molecules.
